# Supplementary material for: Knowledge, attitude, prevention practice and lived experience towards cutaneous leishmaniasis and associated factors among residents of Kutaber district, Northeast Ethiopia, 2022: A mixed method study
Source: PLoS Negl Trop Dis. 2024 Aug 22;18(8):e0012427. doi: 10.1371/journal.pntd.0012427 (PMC11373811; doi:10.1371/journal.pntd.0012427)
Supplement: S1 Questionnaire — (DOCX) [file pntd.0012427.s001.docx]

**English version of the questionnaire**

**Part 1: Questionnaire for quantitative part of the study**

| District: ________ | | House code ____________ | | Kebele: | _________ | Gott: | _________ | |  |
| --- | --- | --- | --- | --- | --- | --- | --- | --- | --- |
| Name of interviewer: | | _______________________ | | Signature: | _________ | Date: | _________ | |  |
| Name of supervisor: | | _______________________ | | Signature: | _________ | Date: | _________ | |  |
| **Socio-Economic and Demographic Characteristics of Respondents (100)** | | | | | | | | | |
| **Code** | **Questions** | | **Response/s** | | | | | **Skip** | |
| 101 | Gender | | 1. Male 2. Female | | | | |  | |
| 102 | How old are you? (in years) | | _______________________________ | | | | |  | |
| 103 | What is your marital status? | | 1. Single 2. Married  3. Divorced 4. Widowed | | | | |  | |
| 104 | What is your educational status? | | 1. Unable to read & write 2. Able to read & write only 3. Literate (__________________________) | | | | |  | |
| 105 | For how long you are resident of this area? | | ____________________________ year/s | | | | |  | |
| 106 | What is your occupation? | | 1. Farmer 2. Housewife 3. Merchant 4. Government employ 5. Student 6. Unemployed 7. Others __________ | | | | |  | |
| 107 | What is the total number of people in the household? | | ____________________________ | | | | |  | |
| 108 | Where is your place of residence | | 1. Rural 2. Urban | | | | |  | |
| **Wealth Indicators** | | | | | | | | | |
| **Rural Wealth Indicators** | | | | | | | | | |
| 109 | Private home Yes….…... 1 No……0 | | | | | | |  | |
| 110 | Type of the house Corrugated iron sheet ….1 thatched roof …….0 | | | | | | |  | |
| 111  112  113  114  115  116  117 | Watch Yes ... 1 No ... 0  Sofa Yes ... 1 No ... 0  Chair Yes ... 1 No ... 0  Table Yes ... 1 No ... 0  Bed and mattress which made from cotton spring Yes ... 1 No ... 0  Horse’s Cart Yes ... 1 No ... 0  If others specify --------------------------- | | | | | | |  | |
| 118  119  120  121  122  123  124  125  126 | Do you have your own farm for agriculture/cropping? Yes ... 1 No ... 0  If yes, Annual farm product (write the amount in the provided space per quintal)  Teff Yes ...1 No ... 0 ---------------- quintal  Barley Yes ...1 No ... 0 ---------------- quintal  Maize Yes ...1 No ... 0 ----------------- quintal  Bean Yes ...1 No ... 0 ------------------ quintal  Onion Yes ...1 No ... 0 ------------------ quintal  Wheat Yes ...1 No ... 0 -------------------quintal  Eucalyptus Tree Yes ...1 No ... 0 _____________(number)  Others, Specify ------------------------------------------ | | | | | | |  | |
| 127  128  129  130  131  132  133 | From the following household animal which one do you have? (More than 1 is possible)  1. Ox/ cow Yes ...1 No ... 0 ------------- (in number)  2. Horse/donkey/ mule Yes ... 1 No ... 0 ------------- (in number)  3. Goat Yes ... 1 No ... 0 ------------- (in number)  4. Sheep Yes ... 1 No ... 0 ------------- (in number)  5. Hen Yes ... 1 No ... 0 ------------- (in number)  6. Beehive Yes ...1 No ... 0 ------------- (in number)  7. Others................... | | | | | | |  | |
| **Urban Wealth Indicators** | | | | | | | | | |
| 134  135  136  137  148  139  140 | What is the source of your drinking water? (More than one answer is possible)  1.houseline water Yes ... 1 No ... 0  2. pull and push/sway common water Yes ... 1 No ... 0  3. bono water Yes ... 1 No ... 0  4.protective pond water Yes ... 1 No ... 0  5.un protective pond water Yes ... 1 No ... 0  6.stream water Yes ... 1 No ... 0  7. if others list ---------------------------------- | | | | | | |  | |
| 141  142  143  144  145 | What type of toilet do you use?  1.Water flush Yes ... 1 No ... 0  2. Traditional toilet Yes ... 1 No ... 0  3. Ventilated improved pit latrine Yes ... 1 No ... 0  4. Open field Yes ... 1 No ... 0  5. Others (list) ………………… | | | | | | |  | |
| 146 | Who is the owner of your living house? 1. My own 0. rent house | | | | | | |  | |
| 147 | Does your living house have dividing class? Yes ... 1 No ... 0 | | | | | | |  | |
| 148 | Do you have separated bedroom? Yes ... 1 No ... 0 | | | | | | |  | |
| 149 | Do you have separated kitchen? Yes ... 1 No ... 0 | | | | | | |  | |
| 150  151  152  153  154 | From which material your house floor is made? (More than one answer is possible)  1. Natural ground Yes ... 1 No ... 0  2. Muck/smooth by cow dung Yes ... 1 No ... 0  3. Wood Yes ... 1 No ... 0  4. Cement Yes ... 1 No ... 0  5. if others list ………………….... | | | | | | |  | |
| 155 | From which material your house roof is made?  0 ……...Grass/ leaf 1………... Corrugated iron | | | | | | |  | |
| 156  157  158  159  160 | From which material your house wall is made? (More than one answer is possible)  1. Wood but not have mud Yes ... 1 No ... 0  2. Wood with mud Yes ... 1 No ... 0  3. Wood and cement Yes ... 1 No ... 0  4. Blocks Yes ... 1 No ... 0  5. if others list…………..…. | | | | | | |  | |
| 161  162  163  164  165  166 | What is your energy source for food cooking? (More than one answer is possible)  1. Electricity system Yes ... 1 No ... 0  2. Gas /kerosene Yes ... 1 No ... 0  3. Wood /leaf Yes ... 1 No ... 0  4. Charcoal Yes ... 1 No ... 0  5. Animal manure Yes ... 1 No ... 0  6. If others, list… | | | | | | |  | |
| 167  168  169  170  171  172  173  174  175  176  177  178  179  180 | Among the following materials, which one do you own? (More than one answer is possible)  1. Radio Yes ... 1 No ... 0  2. Television Yes ... 1 No ... 0  3. House phone Yes ... 1 No ... 0  4. Fridge Yes ... 1 No ... 0  5. Chair Yes ... 1 No ... 0  6. Table Yes ... 1 No ... 0  7. Bed and mattress which made from cotton spring Yes ... 1 No ... 0  8. Mobile Yes ... 1 No ... 0  9. Cycle Yes ... 1 No ... 0  10. Motor cycle Yes ... 1 No ... 0  11. Horse’s cart Yes ... 1 No ... 0  12. Bajaj/car Yes ... 1 No ... 0  13.Bank book Yes ... 1 No ... 0  14. If other, list ……………. | | | | | | |  | |
| **Knowledge Towards CL (200)** | | | | | | | | | |
| **Code** | **Questions** | | **Response/s** | | | | | **Skip** | |
| 201 | Ask the respondent if they could name the disease after showing a picture of CL manifestation. | | 1. Able to identify as CL 2. Unable to identify | | | | |  | |
| 202 | Have you ever heard about CL? | | 1. Yes 2. No | | | | |  | |
| 203 | Have you ever got CL? | | 1. Yes 2. No | | | | |  | |
| 204 | Is CL transmitted by the urine of bats? | | 1. Yes 2. No 3. I don’t know | | | | |  | |
| 205 | Is CL transmitted by the bite of sandfly? | | 1. Yes 2. No 3. I don’t know | | | | |  | |
| 206 | Is skin lesion the sign of CL? | | 1. Yes 2. No 3. I don’t know | | | | |  | |
| 207 | Are face, forehead, nostril, arm, leg and ear the parts of the body for the location of CL lesions/scars? | | 1. Yes 2. No 3. I don’t know | | | | |  | |
| 208 | Are rock crevices, caves, rodent burrows, leaf litters and vegetation  the habitats of sandfly? | | 1. Yes 2. No 3. I don’t know | | | | |  | |
| 209 | Is CL a communicable disease? | | 1. Yes 2. No 3. I don’t Know | | | | |  | |
| 210 | Is there a possibility of acquiring CL in travelling to endemic areas? | | 1. Yes 2. No 3. I don’t Know | | | | |  | |
| 211 | Are dawn and dusk the preferred biting times of the vector? | | 1. Yes 2. No 3. I don’t know | | | | |  | |
| 212 | Is CL a serious disease? | | 1. Yes 2. No 3. I don’t know | | | | |  | |
| 213 | Is CL preventable disease? | | 1. Yes 2. No 3. I don’t know | | | | |  | |
| 214 | Are health education, hygiene, and insecticide the prevention measures for CL? | | 1. Yes 2. No 3. I don’t know | | | | |  | |
| 215 | Is complete cure from CL possible? | | 1. Yes 2. No 3. I don’t know | | | | |  | |
| **Attitude Towards CL (300)** | | | | | | | | | |
| **Code** | **Questions** | | **Response/s** | | | | | **Skip** | |
| 301 | CL is a health problem in your area | | 1. Strongly Agree 2. Agree 3. Neutral 4. Disagree 5. Strongly Disagree | | | | |  | |
| 302 | CL can be treated | | 1. Strongly Agree 2. Agree 3. Neutral 4. Disagree 5. Strongly Disagree | | | | |  | |
| 303 | Disability is the outcome of CL if not treated early | | 1. Strongly Agree 2. Agree 3. Neutral 4. Disagree 5. Strongly Disagree | | | | |  | |
| 304 | The occurrence of CL in one member of the family affects the economy of the whole family | | 1. Strongly Agree 2. Agree 3. Neutral 4. Disagree 5. Strongly Disagree | | | | |  | |
| 305 | Autumn is the season at which the incidence of CL is at its peak/high | | 1. Strongly Agree 2. Agree 3. Neutral 4. Disagree 5. Strongly Disagree | | | | |  | |
| 306 | CL is transmitted by direct contact from person to person | | 1. Strongly Agree 2. Agree 3. Neutral 4. Disagree 5. Strongly Disagree | | | | |  | |
| 307 | Environmental Sanitation is important for prevention of CL transmission | | 1. Strongly Agree 2. Agree 3. Neutral 4. Disagree 5. Strongly Disagree | | | | |  | |
| 308 | You are well informed about CL | | 1. Strongly Agree 2. Agree 3. Neutral 4. Disagree 5. Strongly Disagree | | | | |  | |
| 309 | Vegetation area, rock cracks, termite piles and animal manures are the major breeding places of sandfly | | 1. Strongly Agree 2. Agree 3. Neutral 4. Disagree 5. Strongly Disagree | | | | |  | |
| 310 | CL is spiritual | | 1. Strongly Agree 2. Agree 3. Neutral 4. Disagree 5. Strongly Disagree | | | | |  | |
| 311 | CL has a relation with hyraxes | | 1. Strongly Agree 2. Agree 3. Neutral 4. Disagree 5. Strongly Disagree | | | | |  | |
| 312 | Worrying is the impression of the disease CL | | 1. Strongly Agree 2. Agree 3. Neutral 4. Disagree 5. Strongly Disagree | | | | |  | |
| **Prevention Practice Towards CL (400)** | | | | | | | | | |
| **Code** | **Questions** | | **Response/s** | | | | | **Skip** | |
| 401 | Do you use bed nets? | | 1. Yes 2. No | | | | |  | |
| 402 | When is your work time preference? | | 1. Day time 2. Night 3. Both | | | | |  | |
| 403 | Do you sleep outdoor? | | 1. Yes 2. No | | | | |  | |
| 404 | Do you use repellents for CL Prevention? | | 1. Yes  2. No | | | | |  | |
| 405 | Do you properly perform garbage disposal? | | 1. Yes 2. No | | | | |  | |
| 406 | Has your house ever been sprayed in the last 12 months? | | 1. Yes 2. No | | | | |  | |
| 407 | Have you ever participated in CL control activities? | | 1. Yes 2. No | | | | |  | |
| 408 | What treatment methods do use if you get the disease? | | 1. Modern medicine 2. Traditional medicine 3. Both | | | | |  | |
| **Environmental Factors (500)** | | | | | | | | | |
| **Code** | **Questions** | | **Response/s** | | | | | **Skip** | |
| 501 | What material is the wall surface of the house made from? (Filled by observation) | | 1. Cement 2. Mud 3. Wood   4. Stone 5. Others ____ | | | | |  | |
| 502 | Condition of the wall (filled by the observation) | | 1. No crack/holes formed 2. Cracked  3. Holes formed | | | | |  | |
| 503 | If cracked, which type? (Observation) | | 1. Almost all walls 2. Some of the walls | | | | |  | |
| 504 | What is the type of ceiling/roof? (Filled by the interviewer observation) | | 1. Corrugated iron sheet 2. Straw  3. Others _____ | | | | |  | |
| 505 | What is the type of the floor? (Filled by the interviewer observation) | | 1. Cemented 2. Pasted with cow dung/mud 3. Others ___ | | | | |  | |
| 506 | Do you have latrine? | | 1. Yes 2. No | | | | |  | |
| 507 | The location of the house from creeks or waterways? (Filled by observation) | | 1. Close 2. Not close | | | | |  | |
| 508 | Which one do you use for cooking/heating? | | 1. Wood and charcoal 2. Gas/kerosine 3. Electricity 4. Others … | | | | |  | |
| **Behavioral Factors (600)** | | | | | | | | | |
| **Code** | **Questions** | | **Response/s** | | | | | **Skip** | |
| 601 | Do you have the habit of damping animal dung near house? | | 1. Yes 2. No | | | | |  | |
| 602 | Do you have the habit of working outside at night? | | 1. Yes 2. No | | | | |  | |
| 603 | Do you have the habit of filling cracks and animal burrows? | | 1. Yes 2. No | | | | |  | |
| 604 | Do you have the custom of spending time near/at gorge early morning or night? | | 1. Yes 2. No | | | | |  | |
| 605 | Do you have the habit of openly defecating near or inside forests/ gorges? | | 1. Yes 2. No | | | | |  | |
| 606 | Do you weed round your home? | | 1. Yes 2. No | | | | |  | |
| 607 | Do you open your window at night? | | 1. Yes 2. No | | | | |  | |
| 608 | Have you ever gone to traditional healer? | | 1. Yes 2. No | | | | |  | |
| 609 | Do you use medias? | | 1. Yes 2. No | | | | |  | |
| 610 | Have you received education about CL previously? | | 1. Yes 2. No | | | | |  | |
| 611 | Do you know someone with CL | | 1. Yes 2. No | | | | |  | |

**Thank you for your Participation!**

**Part 2: Questionnaire for Qualitative Part of the study**

1. **In-depth Interview Guiding Questions for Community Residents (Victims)**

| **District** | **_________________** | **Kebele** | **_________________** |
| --- | --- | --- | --- |
| **Date** | **_________________** | **Starting time** | **_________________** |
| **Interviewer** | **_________________** | **Ending time** | **________________** |
| **Interviewee Code** | **_________________** |  |  |

1. Gender of the interviewee_____________________________________
2. Age of the interviewee________________________________________
3. Marital status of the interviewee ________________________________
4. Occupation of the interviewee __________________________________
5. How do you perceive the severity of cutaneous leishmaniasis? Who are more affected by this disease? What action does the community take if someone is infected by cutaneous leishmaniasis? How we behave in general with those affected by cutaneous leishmaniasis? Why people stigmatize those who are affected by the disease?
6. What are the perceived barriers for implementing preventive measures against cutaneous leishmaniasis?
7. Where did this community obtain water? At what time the community mostly collect the water?
8. Why people are going to forests/vegetations? At what time they mostly go to the forest??
9. Where do you, your family and community members would like to take part for recreation? At what time mainly?
10. Where do you see hyraxes in the community? Why and when people are going to hunt them?
11. What do you know about traditional healers? What is their role in treating cutaneous leishmaniasis? What are the reasons for visiting them rather than health care providers?
12. What kind of medias are available here for the dissemination of health information including information about cutaneous leishmaniasis? Where did you get information about cutaneous leishmaniasis? What roles did healthcare facilities & schools played in health education & information dissemination about cutaneous leishmaniasis and its prevention?
13. What the community generally does to prevent cutaneous leishmaniasis? What personal protective equipments are available to do so? How do they use them?

**Thank you for your participation!**

**Questionnaire for Qualitative Part**

1. **In-depth Interview Guiding Questions for Key Informants**

| **District** | **_________________** | **Kebele** | **_________________** |
| --- | --- | --- | --- |
| **Date** | **_________________** | **Starting time** | **_________________** |
| **Interviewer** | **_________________** | **Ending time** | **________________** |
| **Interviewee Code** | **_________________** |  |  |

1. Gender of the interviewee____________________________________
2. Age of the interviewee_______________________________________
3. Marital status of the interviewee _______________________________
4. Occupation of the interviewee _________________________________
5. What do you know about cutaneous leishmaniasis of this community?
6. How do you perceive the severity of cutaneous leishmaniasis in this community? Who are more affected by the disease? What actions that you know does the community take if someone is infected by cutaneous leishmaniasis? Why the community stigmatize the victims?
7. What are the perceived barriers for implementing preventive measures, active case detections and treatment options of cutaneous leishmaniasis?
8. What are the means of collaborations made and going on between traditional healers of the community and health care facilities in treating cutaneous leishmaniasis? What are the barriers for the collaboration?
9. What kind of medias are available and most frequently used for information communication about cutaneous leishmaniasis in this community? What is your and your organization’s effort in disseminating health information about the disease?
10. What you and your organization generally did and will do to prevent cutaneous leishmaniasis?

**Thank you for your participation!**
