# Supplementary material for: Knowledge, attitude, prevention practice and lived experience towards cutaneous leishmaniasis and associated factors among residents of Kutaber district, Northeast Ethiopia, 2022: A mixed method study
Source: PLoS Negl Trop Dis. 2024 Aug 22;18(8):e0012427. doi: 10.1371/journal.pntd.0012427 (PMC11373811; doi:10.1371/journal.pntd.0012427)
Supplement: Attachment — Submitted filename: Letter of Response to Editors and Reviewers.docx [file pntd.0012427.s003.docx]

**15/06/2024**

**To Esteemed Editors and Reviewers**

**Subject: Response to Editors’ and Reviewers’ Comments and Suggestions**

It is our great pleasure to thank the editors and reviewers of our manuscript entitled **“Knowledge, attitude, prevention practice and lived experience towards cutaneous leishmaniasis and associated factors among residents of Kutaber district, Northeast Ethiopia, 2022: a mixed method study”** for the valuable comments and/or suggestions. Upon conducting a comprehensive review of the comments, suggestions and questions provided and asked, we have meticulously addressed each one. We are confident that the revisions we’ve made meet your expectations and significantly improve the quality of our manuscript. To aid in the review process, we have prepared two versions of the manuscript for your convenience: one with clear, easy to read copy and the other highlighted one showing changes made from previously submitted version of the manuscript. These versions aim to facilitate understanding and transparency regarding the modifications made. We are pleased to present the revised manuscript along with our responses to the comments. We eagerly await your favorable consideration for publication.

**With Regards!**

**Authors**

**Part-I: Authors Response to Editor(s)’ Comments/Suggestions**

| **Editor(s)’ Comments/Suggestions to Authors** | | **Authors’ Response** |
| --- | --- | --- |
| **#1** | I invite you to respond to the reviewers’ comments and revise your manuscript. We cannot make any decision about publication until we have seen the revised manuscript and your response to the reviewers' comments. Your revised manuscript is also likely to be sent to reviewers for further evaluation | Thank you for the invitation to respond to the reviewers' comments and revise the manuscript. We have thoroughly addressed the suggestions and are pleased to submit the revised version for your consideration. |
| **#2** | When you are ready to submit, please upload a letter containing a detailed list of your responses to the review comments and a description of the changes you have made in the manuscript. Please note while forming your response, if your article is accepted, you may have the opportunity to make the peer review history publicly available. The record will include editor decision letters (with reviews) and your responses to reviewer comments. If eligible, we will contact you to opt in or out. | Thank you for your guidance. In a response to the review comments and questions, a description of the changes that we have made in the manuscript was well-prepared. We are also volunteer in making the peer review history publicly available if our article is accepted. |
| **#3** | When you are ready to submit, please upload two versions of the revised manuscript: one with either highlights or tracked changes denoting where the text has been changed; the other a clean version (uploaded as the manuscript file). | Thank you for the guiding suggestion. We highlighted the Microsoft word document in red colour to show where the changes made in the manuscript. Moreover, we have prepared another clean version (uploaded as the manuscript file). |
| **#4** | Please prepare and submit your revised manuscript within 60 days. If you anticipate any delay, please let us know the expected resubmission date by replying to this email. Please note that revised manuscripts received after the 60-day due date may require evaluation and peer review similar to newly submitted manuscripts | Thank you for your reminder and consideration. Due to the state of emergency declared in Ethiopia, the interruption of internet service was continuing. In this case, we anticipated a delay and asked a due date extension for the submission of the revised manuscript. We benefited more from the extension of the deadline. |
| **#5** | While revising your submission, please upload your figure files to the Preflight Analysis and Conversion Engine (PACE) digital diagnostic tool, [https://pacev2.apexcovantage.com](https://pacev2.apexcovantage.com/).  PACE helps ensure that figures meet PLOS requirements. To use PACE, you must first register as a user. Then, login and navigate to the UPLOAD tab, where you will find detailed instructions on how to use the tool. If you encounter any issues or have any questions when using PACE, please email us at [figures@plos.org](mailto:figures@plos.org). | Thank you for your helpful guidance. We have uploaded and ensured our figures met PLOS requirements by using the Preflight Analysis and Conversion Engine (PACE) digital diagnostic tool. |
| **#6** | Please note that, as a condition of publication, PLOS' data policy requires that you make available all data used to draw the conclusions outlined in your manuscript. Data must be deposited in an appropriate repository, included within the body of the manuscript, or uploaded as supporting information. This includes all numerical values that were used to generate graphs, histograms etc. | Thank you. We are pleased to make all the required data used to draw the conclusions outlined in the manuscript available. |

**Part-II: Authors Response to Reviewers’ Comments/Suggestions**

|  | **Contents** | **Review Questions** | **Comments/Suggestions and Questions** | **Authors’ Response** |
| --- | --- | --- | --- | --- |
| **#1** | **Abstract** |  | Rephrase the results section to be more compelling with better clarity (not necessary to report CI's in abstract. i.e. "The survey respondents in Kutaber district showed good knowledge of (47.5%), a positive attitude towards (54.1%) and good prevention practices (35.3%) regarding cutaneous leishmaniasis" | Thank you for your valuable comments. It is thoroughly revised and corrected in the manuscript (i.e. the confidence intervals are removed). |
|  |  |  | It would be more informative if definitions for what good and positive mean. If 50% was a pass, then 47.5% and 35.3% would not be classified as "good". What is a positive attitude - is it positive because the value exceeded 50%? | Thank you. This is clearly stated under the operational definitions and measurements section of the manuscript. The classification was made using mean score of the overall respondents. Respondents who scored below the mean result were classified as having poor knowledge and prevention practice and respondents who scored equal to and above the mean score were classified as having poor knowledge and prevention practice about and towards cutaneous leishmaniasis. Similarly, mean score was used for classifying the attitude of respondents. |
| **#2** | **Introduction** |  | Should logically flow from a global scene to something specific to this research. line 53 starts to discuss Ethiopia and then by line 57 jumps back to the global situation of CL. | Thank you. We have thoroughly reviewed and addressed it. |
|  |  |  | CL has been known since 1913" - too vague. was known by who? local populations? was it introduced as a disease in 1913 or has been endemic for much longer? | We sincerely appreciate the reviewer's feedback and we addressed the comments following a thorough review. |
|  |  |  | Explain why only the highland areas are endemic - is there something special about the highland regions (climate/geography/flora/fauna/how people live) or is it simply there is no data from other regions to compare to? Should discuss the cycle of leish in this region, especially as your survey questions mention rock hyrax and bats. | Thank you. This is because the intermediate hosts (rock hyraxes), the species of the disease vector (sandfly species) are commonly found and intertwined in the dense-forested highland areas of the regions. |
|  |  |  | what were the gaps in prevention methods reported? what prevention methods were used? | Thank you. We have addressed these questions in the manuscript. |
|  |  |  | why is CL a growing concern in the Amhara region? | Thank you for your insightful question. This is thoroughly corrected in the manuscript. |
|  |  |  | L81, 82, 83 and elsewhere - be aware of the overuse of the word "conducted" | Thank you for your positive feedback. It is corrected in the manuscript. |
|  |  |  | why is it important to capture "lived experience" and what do you mean by this? | Thank you. This is addressed in the operational definition and measurement section of the manuscript. |
|  |  |  | what will all this data be used for / contribute to in the future? is there evidence from other CL areas or other diseases in Ethiopia that such knowledge is important? | Thank you. This is addressed in the introduction section of the manuscript. The results of this study will help policy-makers, healthcare planners, and other concerned bodies to plan and implement strategies to create awareness, prevent, and control CL. |
| **#3** | **Methods** | Are the objectives of the study clearly articulated with a clear testable hypothesis stated?  Is the study design appropriate to address the stated objectives?  Is the population clearly described and appropriate for the hypothesis being tested?  Is the sample size sufficient to ensure adequate power to address the hypothesis being tested?  Were correct statistical analysis used to support conclusions? | Methods are well designed to answer the questions raised by the study. My concern is about the implementation of the questionnaires. It is not clear if the questions were adapted to the cultural and social aspects of the communities involved. In my opinion this is of upmost importance to get reliable information. The authors should explain if it was done and how. In case this adaptation was not done, authors should discuss this important limitation. | Thank you for your feedback. The questions were adapted from different literatures to the socio-cultural aspects of the community. We have done it by thorough review of existing literatures. |
|  |  |  | This manuscript addresses an interesting and valuable multifaceted question: how do people in Northeast Ethiopia understand, cope with, treat and prevent cutaneous leishmaniasis in 2022?  The authors have compiled a vast amount of data, both quantitative and qualitative, and from this, have drawn conclusions according to the responses of 612 survey respondents and from 20 in depth interviews with patients and key informants.  Although the manuscript contains a wealth of information, the section organization, data presentation and validation, and deeper data interpretation is lacking.   Consequently, to publish this work with PLoS NTD will require a major rewrite. | Thank you for your important comments and suggestions. We have revised our manuscript and thoroughly corrected them. |
|  |  |  | Below are some of the areas that need additional revision and I have introduced several comments that, if addressed, will make it easier for future readers to understand the significance of this work. | Thank you very much. Below are the responses for the areas that need additional revision and the comments and questions addressed to make it easy for future readers to understand the significance of this scientific work. |
|  |  |  | Explain in detail why the Kutaber district was selected as the study site for this work. | The selection of Kutaber district was done based on the occurrence and burden of cases registered at the dermatology department of Boru-Meda Hospital between 2016 and 2021 along with the ecology of the district. |
|  |  |  | Those "who have experienced CL" means of family or personally? in what time frame? how were the cases documented - via the community, healers or hospital records? | It implies those who have experienced CL personally. |
|  |  |  | What were the inclusion and exclusion criteria used to define the study populations for both the quantitative and qualitative studies? | The inclusion and exclusion criteria used for both qualitative and quantitative study is clearly described under the inclusion criteria section of the revised manuscript. The confirmed cases were documented by the hospital using leishmaniasis registration book. |
|  |  |  | Explain why the scoring was based on the mean scores.  was this overall or per the sampling sites Kutaber, Alansha, Doshign, Beshilo, Haroye and Kundi? | Since there were no literatures having clear-cut classifications and some were used mean score for classification, we used the mean to classify the respondents for having good/poor knowledge about and prevention practice of cutaneous leishmaniasis. The mean was calculated for the overall participants of the study. |
|  |  |  | It is not enough to simply know bed net utilization - what type of bed nets did people have access to?  many bed nets are for malaria prevention and consequently the weave is not small enough to prevent sand flies from biting.   the lack of using a bed net, regardless of the presence of holes, for CL could indicate the person knows it cannot protect against biting sand flies! | Regarding bed nets, there are evidences that stated the distribution of insecticide-treated net and house spraying to prevent malaria have a positive impact on the control of sand flies. |
|  |  |  | What is house spray? | Thank you very much. House spray is the indoor residual spray of the home environment twelve months before data collection. |
|  |  |  | What CL control and treatment options were available in this district? | From an in-depth interview result, traditional treatment options were the most widely practiced one in the district. From the quantitative result, the figure for traditional treatment is not that much significantly different even though modern treatment was found to be their preference to use. |
|  |  |  | Further describe the parameters used to assess household wealth and explain why some of those parameters were not used in Kutaber. | The parameters used to assess household wealth were taken from the one which was nationally harmonized and used in country. |
|  |  |  | What language was used for the questionnaire? were translators used? | Amharic language was used for the questionnaire and language translators were used. |
|  |  |  | Where is the data stored (including signed informed consent sheets) and how long will data be stored before it is destroyed? | The data were stored and locked in the file cabinet. The data would be stored for three years and more before it is properly destroyed. |
|  |  | Are there concerns about ethical or regulatory requirements being met? | There are no regulatory or ethical concerns. | We thank you. |
| **#4** | **Results** | Does the analysis presented match the analysis plan?  Are the results clearly and completely presented? | The analysis of the survey results are highly confusing and where survey questions were repeated, it is not clear why different responses were recorded.  The tables, as they are presented are fractionated and more applicable for a thesis style of writing.   It would be far more informative to have all the responses to each question in a single table, with colour coded sections to represent each section.  Suggest also revisiting all the data as the text does not match what is in the tables, and some of the tabulated data do not agree when it should. | Thank you for your insightful comments. We thoroughly revise and corrected it in the manuscript. |
|  |  |  | Explain what different types of media you assessed the usage of for scoring. | The types of medias we assessed for the usage were radio, television, microphone and social medias (Facebook, telegram, and YouTube). |
|  |  |  | "Knew someone with CL" - at the time of the survey this was someone with an active case or rather at some point in the interviewee's life, they had encountered someone with CL? | This was at some point in the interviewee's life, they had encountered someone with the disease CL. |
|  |  |  | Explain what prompted the questionnaire question of CL transmission through bat urine? is it because of caves in the area? or behaviors? or a legend? | It’s because of the ecological nature of the area and suggestions from experts involved in the expert judgement. |
|  |  |  | Table 2: Question: Is CL a communicable disease? Communicable is considered a health jargon word. How did you confirm that persons answering this question understood what it meant in the way you wanted them to? | Thank you. This is corrected clearly as ‘is CL a disease transmitted from an infected person to a healthy person?’ |
|  |  |  | It is troubling to see that the numbers described in "attitude towards CL" section do not match what is presented in the tables. i.e., "About 378 of the study participants agreed the CL is a health problem in their area, but about 75 of the participants disagreed with this idea". According to the referenced Table 3, only 278 patients agreed or strongly agreed with this statement and 230 disagreed with this idea. This mismatch between table and text is throughout this section and other places in the results. | Thank you for highlighting the mismatch between the table and the text. This is assessed and thoroughly corrected in the manuscript. |
|  |  |  | Participants preferred modern medicine over what? | Thank you. Participants preferred to use modern medicine over traditional or a combination of modern and traditional treatment if they get the disease CL. |
|  |  |  | Some sections of Table 5 and 6 are not in logical agreement. Considering the same participants are completing the survey at a single timepoint, it is not explained why answers differ between the two tables when certain stats are recorded like years of residence. Are the tables showing that how long a person lives in this district affects their knowledge of CL (Table 5) or their attitude towards CL (Table 6) in opposite ways (especially if participants have only been there for 1 -2 years? This is interesting as it shows that knowledge of CL is poor in this group yet their attitude towards CL can remain positive? This and other such observations need to be further expanded in the discussion. | Thank you. The quantitative part of the study has three different dependent variables (i.e., Knowledge, Attitude and Prevention practice). All of the dependent variables have their own cross-tab results and this might not be expected to have exactly the same result. |
|  |  |  | Expand on what metrics you used to assess physical, social, psychological impacts and whether body language / speech / emotional inputs were also assessed during interviews. | Thank you. Inputs from speech and body language were thoroughly assessed during an in-depth interview. |
|  |  |  | It would be helpful for the reader to understand what some of the quotations are referring to i.e., what does a "cut nose" mean? Is this a treatment? Such powerful quotes you have captured! | Thank you for your insightful comments and questions. It is thoroughly corrected in the manuscript. |
|  |  |  | Priority was given to traditional healers. How did this compare to the data collected from the questionnaire? | Thank you. From an in-depth interview, it was found that traditional treatment was the preferred treatment method in the district. It is supported by the quantitative finding even though a very little difference was recorded between the treatment methods. |
|  |  | Are the figures (Tables, Images) of sufficient quality for clarity? | Fig1.  a) Label the different maps in the order you want the reader to look at them.   By default, an English reader will read from left to right so will naturally look at the blue map first and then the pink one, which is incorrect if the point is to move from larger to smaller geographical scale. b) For the first blue panel, please add in the many countries bordering Ethiopia so it is easier to locate the study region and understand its position on the continent.  Include a marker for Addis as you mention it in your M&M. c) Add in map scale to the other 3 panels d) If you highlight the Kutaber District on the orange Amhara Region panel, the pink map panel can be removed. e) It is more informative to add in some geographical features to the Kutaber Woreda map such as rivers, lakes, major cities (Kutaber, Dessie etc), deserts, mountains, and primary roadways | Thank you for your comment. We have taken the shapefiles of the map from the department of Geographic Information System, Wollo University. But we couldn’t get the original source and link for it. Finally, we decided to remove the map from the manuscript. |
|  |  |  | Fig3.  On  my printout, this figure is impossible to read. a) Please ensure everything has been standardized to the same quality, size and font. b) change black lettering on dark green or purple bottom squares to a white font c) need to incorporate directionality to the image = what do you want the reader to read first, second etc... d) the font size on the branches / lines must be increased or if not enough room, label each line with a number and describe it in the figure legend | Thank you very much for your insightful comments. We have revised our manuscript thoroughly and checked our figure files to the Preflight Analysis and Conversion Engine (PACE) digital diagnostic tool. We have thoroughly revised and corrected them and the figure for each of the five themes are prepared separately. |
| **#5** | **Discussions** |  | Consider tabulating the prioritized data you have mentioned so the discussion is not re-iterating data presented in the results and rather explain why these trends are appearing and what this means for future disease management. | Thank you. We have revised the results section of the manuscript well and aligned it with the discussion part of the manuscript. |
|  |  |  | What are the traditional treatment side effects?  Why do more people continue to seek out a healer when they see that friends/family are not being healed, especially if there are side effects to this treatment? Is it simply a fear of injections that drives this behavior or something else? | Thank you for your questions. The experienced traditional treatment side effects were disease complication, delays of healing, and scar formation. The reason for the continued seeking of traditional treatment were the affordability of traditional herbs, fear of injection, deep-rooted cultural belief in its effectiveness, and long waiting times at healthcare facilities. |
| **#6** | **Conclusions** | Are the conclusions supported by the data presented? | The conclusions may be biased by a possible lack of cultural and social integration of the tools used to evaluate some factors that were investigated. Authors should discuss this limitation and possible interference on the interpretation of the data collected. | Thank you for your insightful comments. We have thoroughly discussed the issue and addressed well in the manuscript. |
|  |  | Are the limitations of analysis clearly described? | Not commented | The limitations of the study were clearly described in the manuscript. |
|  |  | Do the authors discuss how these data can be helpful to advance our understanding of the topic under study? | All of these conclusions need to be revisited by the authors. The data requires correction, the study details need to be expanded (i.e., what were the exclusion / inclusion criteria for the participants?) | Thank you for your insightful comments. We have thoroughly revised our manuscript. |
|  |  | Is public health relevance addressed? | What knowledge gap is this data filing and how will it be used in the future, how will this study improve the leishmaniasis knowledge, treatment, prevention of the residents of Kutaber district? | Thank you very much. The public health relevance of the study is clearly addressed in the manuscript. |
| **#7** | **Summary and General Comments** |  | As outlined in the previous sections, I have suggested areas that need further clarification, information or correction.   With a serious rewrite, this manuscript will provide valuable information to decision makers in public health, as to where resources for CL should be prioritized - i.e. prevention, education or treatment.   The quotes captured during the in-depth interviews is insightful and clearly shows how keen those living in this district are to better understand and prevent CL | Thank you. We thoroughly revised and rewrite the manuscript. |
|  |  |  | The recommendation of major revision does not require new experiments, but rather a better analysis of the data. | Thank you. We analyzed and presented the data thoroughly. |
|  |  |  | As participants from six different Kebeles completed the surveys, there should be a comparison between them (or a strong explanation why not), to understand if CL perceptions were conserved across the households, or whether there were certain foci that held strongly to different knowledge, attitudes and practices. | Thank you. This is included under the limitation of the study section of the manuscript. |
