# Supplementary material for: Knowledge, attitude, prevention practice and lived experience towards cutaneous leishmaniasis and associated factors among residents of Kutaber district, Northeast Ethiopia, 2022: A mixed method study
Source: PLoS Negl Trop Dis. 2024 Aug 22;18(8):e0012427. doi: 10.1371/journal.pntd.0012427 (PMC11373811; doi:10.1371/journal.pntd.0012427)
Supplement: Attachment — Submitted filename: A Letter of Response to Editors and Reviewers.docx [file pntd.0012427.s004.docx]

**20/07/2024**

**To Esteemed PLOS NTD Editors and Reviewers**

**Subject: Response to Editors’ and Reviewers’ Comments and Suggestions**

It is our great pleasure to thank the editors and reviewers of our manuscript entitled **“Knowledge, attitude, prevention practice and lived experience towards cutaneous leishmaniasis and associated factors among residents of Kutaber district, Northeast Ethiopia, 2022: a mixed method study”** for the crucial and valuable comments and/or suggestions. Upon conducting a comprehensive review of the comments and suggestions provided, we have meticulously addressed each of them. We are confident that the revisions we have made meet your expectations and significantly improve the quality of our manuscript too. To aid in the review process, we have prepared two versions of the manuscript for your convenience: one with clear, easy to read copy and the other tracked one showing changes made from previously submitted version of the manuscript. These versions aim to facilitate understanding and transparency regarding the modifications we have made. We are pleased to present the revised manuscript along with our responses to the comments and suggestions. We eagerly await your favorable consideration for publication.

**With Regards!**

**Authors**

**Part-I: Authors Response to Editor(s)’ Comments/Suggestions**

| **Editor(s)’ Comments/Suggestions to Authors** | | **Authors’ Response** |
| --- | --- | --- |
| **#1** | Note from editor: the manuscript has significantly improved, but please address all issues clearly described by both reviewers. Some of these were already pointed out by the reviewers and not properly addressed in the revised version. | Thank you very much for your suggestions and invitation to respond to the reviewers' comments and revise the manuscript. We have thoroughly addressed the suggestions and are pleased to submit the revised version for your consideration. |
| **#2** | When you are ready to submit, please upload a letter containing a detailed list of your responses to the review comments and a description of the changes you have made in the manuscript. Please note while forming your response, if your article is accepted, you may have the opportunity to make the peer review history publicly available. The record will include editor decision letters (with reviews) and your responses to reviewer comments. If eligible, we will contact you to opt in or out. | Thank you once more for your valuable guidance. In a response to the review comments and suggestions, a description of the changes that we have made in the manuscript was well-prepared. We are also volunteer and pleased in making the peer review history publicly available if our article is accepted. |
| **#3** | When you are ready to submit, please upload two versions of the revised manuscript: one with either highlights or tracked changes denoting where the text has been changed; the other a clean version (uploaded as the manuscript file). | We thank you for the guiding suggestions. We have prepared a tracked Microsoft word document showing where the changes made in the manuscript. Moreover, we have prepared another clean version (uploaded as the manuscript file). |
| **#4** | Please prepare and submit your revised manuscript within 30 days. If you anticipate any delay, please let us know the expected resubmission date by replying to this email. | Thank you for your reminder and consideration. We do not have any anticipation for a delay in thoroughly revising the manuscript. |
| **#5** | References: please review your references list to ensure that it is complete and correct. If you have cited papers that have been retracted, please include the rationale for doing so in the manuscript text., or remove these references and replace them with relevant current references. Any changes to the reference list should be mentioned in the rebuttal letter that accompanies your revised manuscript. If you need to cite a retracted article, indicate the article’s retracted status in the references list and also include a citation and full references for the retraction notice. | Thank you very much again. We have thoroughly reviewed our references list and ensured that it is complete and correct. We do not cited papers that have been retracted. Moreover, we do not have any changes with the references list. |

**Part-II: Authors Response to Reviewers’ Comments/Suggestions**

|  | **Contents** | **Comments/Suggestions and Questions** | **Authors’ Response** |
| --- | --- | --- | --- |
| **#1** | **Methods** | Very well done, methodology seems adequate and well designed to obtain fine data and interpretation. However, the outputs “Good knowledge versus poor knowledge”, “Attitude” and “Prevention Practice” should not be dichotomic. The difference between those near the mean score is very narrow, its accuracy may be lower than desirable in a so complex reality. I think that it is important to modify it, and establish a third output, for example: “Regular Knowledge” (also for others). Another option would be excluding them from the outputs those who are near the mean score (for example: 5 % above or low). | Thank you very much for your insightful comments.  We have done the dichotomization through a thorough review of literatures. We have discussed the issue of the dichotomization and addressed into the limitation part of the manuscript too. |
| **#2** | **Results** | I would like to suggest that three of the rebuttal comments are embedded into the manuscript as they give valuable added insight to the reader.  Q. It is not enough to simply know bed net utilization - what type of bed nets did people have access to?  many bed nets are for malaria prevention and consequently the weave is not small enough to prevent sand flies from biting.   the lack of using a bed net, regardless of the presence of holes, for CL could indicate the person knows it cannot protect against biting sand flies!  A. Regarding bed nets, there are evidences that stated the distribution of insecticide-treated net and house spraying to prevent malaria have a positive impact on the control of sand flies.  What are these EVIDENCES? REFERENCES? | Thank you very much once more for your insightful comments.  The evidences stated that the distribution of ITN and indoor residual spray (IRS) or house spray have a positive impact on the significant reduction of sandfly densities.  It was clearly presented on the result section (Page 4 and 5) of the first reference placed below. Moreover, on the discussion part, it was clearly stated verbatim as: “Overall, our results indicated that the IRS/LLINs has had an impact on the sand fly vector density and as a result on the incidence of CL in humans.”  **The references are:**   1. Coulibaly, C. A., Traore, B., Dicko, A., Samake, S., Sissoko, I., Anderson, J. M., ... & Doumbia, S. (2018). Impact of insecticide-treated bed nets and indoor residual spraying in controlling populations of Phlebotomus duboscqi, the vector of Leishmania major in Central Mali. *Parasites & vectors*, *11*, 1-7. 2. Alexander, B., & Maroli, M. (2003). Control of phlebotomine sandflies. *Medical and veterinary entomology*, *17*(1), 1-18. |
| **#3** | **Conclusions** | I agree with the conclusions and limitations presented. However, a better analysis of the outputs “Good knowledge versus Poor Knowledge”, “Attitude” “Prevention Practice” should be provided. | Thank you very much for your insightful comments. We have thoroughly revised our manuscript and addressed it. |
|  |  | In your conclusions, now that you have assessed the data, it would be highly valuable to add your own data-driven perspective on what would be the best specific practice moving forward as follow on from your las sentence. “The finding of this study implied that there is a need for intervention in terms of rigorous provision of health education and implementation of prevention strategies”  Expand on your ending sentences above: “improved health education” TO WHOM (healthcare workers, healers, communities, patients) and elaborate on the prevention STRATEGY WOULD BE THE MOST EFFECTIVE to prevent CL transmission in this region? If you want this data be influential, create signposts for those seeking impactful advice on CL management, whether it be community members, doctors, politicians, health sector. | Thank you very much for your insightful comments. We have thoroughly reviewed the manuscript and addressed these important issues clearly. |
| **#4** | **Editorial and Data Presentation Modification** | Changes to the manuscript are well received however there are a few items that need further attention as described. | Thank you very much again. We have addressed them after thoroughly reviewing our manuscript. |
| **#5** | **Summary and General Comments** | The authors have invested a great amount of time in responding to my suggestions and revising the manuscript. It has improved the context of the work and the quality of the assessment, and the back ground of CL in Ethiopia is better described. | We thank you very much. |
